# Knowledge, attitudes and practice regarding environmental friendly disinfectants for household use among residents of China in the post-pandemic period

**DOI:** 10.3389/fpubh.2023.1161339

**Published:** 2023-04-13

**Authors:** Yongxin Tong, Zerong Zhu, Wenjing Chen, Fang Wang, Xianmin Hu, Jun Wang

**Affiliations:** ^1^Hubei Province Key Laboratory of Occupational Hazard Identification and Control, College of Medicine, Wuhan University of Science and Technology, Wuhan, China; ^2^Institute of Pathogenic Biology, Wuhan Centers for Disease Prevention and Control, Wuhan, China

**Keywords:** environmental friendly disinfectants, emerging contaminants, knowledge, attitudes, practice, residents of China, COVID-19

## Abstract

**Background:**

A sharp rise in household consumption of disinfectants triggered by COVID-19 pandemic has generated tremendous environmental burden and risks of disinfectant emissions in the post-pandemic period. To address this emerging challenge, replacing highly hazardous disinfectants with more environmental friendly alternatives has been accepted as an inherently effective solution to environment issues posed by disinfectant emerging contaminants. However, no study has yet been done to explore the potential customers' attitudes and the market prospect of environmental friendly disinfectants until now.

**Methods:**

This cross-sectional questionnaire-based survey was conducted from January to March, 2022, among resident volunteers in China, to explore the practices, knowledge and attitudes of the public regarding environmental friendly disinfectants for household use.

**Results:**

Among a total of 1,861 Chinese residents finally included in the analyses, 18% agreed or strongly agreed that they paid special attention to the environmental certification label on the product, and only bought the environmental certified disinfectant products; 16% and 10% were using environmental friendly disinfectants for hand sanitization and environmental disinfection, respectively. The mean self-assessed and actual knowledge scores were 2.42 ± 1.74 and 2.12 ± 1.97, respectively, out of a total of 5. Participants having good practices of consuming environmental friendly disinfectants achieved higher knowledge scores. Residents' overall attitudes toward the development, consumption and application of environmental friendly disinfectants were very positive. “*Possible conflict between disinfection effectiveness and environmental factor of disinfectants in a context of severe COVID-19 pandemic*” was considered as the most important barrier jeopardizing the participants' usage intention for environmental friendly disinfectants.

**Conclusions:**

These data suggested most residents of China had a positive attitude, poor knowledge and practices toward environmental friendly disinfectants. More should be done to enhance the residents' environmental knowledge levels about disinfectants, and to further develop and promote disinfectant products with both excellent disinfection activity and environmentally friendly attributes.

## 1. Introduction

During the COVID-19 pandemic, the excessive use of disinfectants in different settings including healthcare, household and public places as well as the intensified disinfection of wastewater result in the increasing environmental loads of disinfectants as emerging contaminants (1–5). Wastewater surveillance testing had shown a strong positive association between the total concentrations of disinfectants and the SARS-CoV-2 viral load in wastewater (4). After being consumed and used for disinfection, disinfectants would be washed down the drain at household and hospital levels, be released during product manufacturing, or run off into stormwater systems after outdoor application (5). Especially within the home-based setting, disinfectants such as benzalkonium chlorides and sodium hypochlorite are widely consumed by the residents as active ingredients in personal care and daily chemical products including hand sanitizers, cleaning agents, disinfection sprays, band-aids, sanitary wipes and wood preservatives, etc., (2). The significant increase of public consumption for household disinfectant products triggered by COVID-19 pandemic greatly contributes to the tremendous environmental burden of disinfectant emissions in the post-pandemic period (2). Growing evidences have demonstrated various health, environmental, and ecological hazards associated with the residue and by-product of various common disinfectants, being typically represented by chlorine-based disinfectants (6) and quaternary ammonium compounds (7, 8), at the environmentally relevant concentrations. For example, exposure to humidifier disinfectants, such as polyhexamethylene guanidine and benzalkonium chlorides, has been identified as the cause of a social disaster in Korea, 2011, which led to more than hundreds of deaths among pregnant women and infants from pulmonary diseases (8). Ecotoxicity data for disinfectant quaternary ammonium compounds suggested their persistent, bioaccumulative and toxic (PBT) potentials at various trophic levels, including algae, fish, invertebrates, and aquatic plants (7). Chlorine-based disinfectants are harmful to aquatic organisms through directly damaging cell walls or proteins by oxidation. Moreover, considerable chlorine disinfection by-products are carcinogenic to humans, and their occurrence in the water environment has adverse effects on the aqueous flora and fauna, as well as microorganisms and plankton present in the ecosystems (1, 6, 9). In view of their preservative and biocidal properties, disinfectants might “wipe” some sensitive commensals in environmental niches and reduce the diversity of microorganism community (10). Therefore, the excessive environmental loads of disinfectants in response to COVID-19 pandemic have been as a cause for worldwide secondary disasters based on their threats to the health of the aquatic ecosystem and sustainability of biodiversity (9–11). Accordingly, how to minimize the risks posed by disinfectant emerging contaminants is of global concern and has emerged as a challenge for modern ecological sustainability in the post-pandemic period (11).

In order to effective combat the ongoing COVID-19 pandemic, the use of disinfectants plays an essential role in guarding against SARS-CoV-2 transmission and infection waves. Under such circumstance, in addition to rational use of disinfectants complying with recommended guidelines, replacing highly hazardous disinfectants with more environmental friendly alternatives has been accepted as an inherently effective solution to the environment issues posed by disinfectant emerging contaminants (3). Despite the fact that there is still no exact definition of the term “environmental friendly disinfectants”, it has been generally accepted that disinfectants being relatively safe to the environment are mainly characterized by the following two aspects. On the one hand, an environmental friendly disinfectant can be fast degraded in the environment and its degraded residuals are non-toxic to organisms (5, 12). Among the common chemical disinfectants, the hydrogen peroxide would readily break down into oxygen and hydrogen within a short period under natural conditions, thus highly unstable in environment (3, 5). Based on their high likelihood of quickly evaporating following application, the volatile disinfectants such as alcohols, bromochloromethane, bromoethane, and chlorine dioxide are unlikely to reach the environmental matrices (3). In recent years, some novel eco-friendly chemical disinfectants, such as phthalocyanin-substituted ammonium compound (12), acidic electrolyzed water (13), commercially available disinfectants Benefect^®^ (14) and Clyraguard copper iodine complex (15), etc., have been developed and demonstrated to be effective for pathogen inactivation, more importantly, not to cause secondary hazard to the environment after administration. On the other hand, more and more researchers focus interest on the development of natural green disinfectants extracted from natural herbs and microorganisms, as the safer and reliable alternatives to the toxic chemical disinfectants for health and the environment (16, 17). So far, numerous medicinal plants being rich in alkaloids, flavonoids, poly-phenolic compounds have been demonstrated to possess authenticated antiseptic, antimicrobial and disinfectant properties with zero toxic impact on environment (17). Especially for the fight against COVID-19 pandemic, considerable non-toxic natural products including tea tree oil (16), coffee leaf extract (18), proanthocyanidins (19), etc., have been reported to possess the potential to be added for hand sanitizers, disinfection sprays and other household disinfectant products to effectively avoid SARS-CoV-2 infection. Moreover, plant-derived natural disinfectants not only are environmentally friendly, but also often act by new action mechanisms that can overcome the developed resistance, thus being considered as a class of emerging and promising disinfectant candidates (20).

Although the environmental friendly disinfectants have been a hotspot in research, no study has yet been done to explore the potential customers' attitudes and the market prospect of this emerging class of disinfectants until now. Therefore, the present study conducted a survey among the residents of China to understand the public's knowledge, attitudes, and practice toward environmental friendly disinfectants for household use during the current post-pandemic period.

## 2. Methods

### 2.1. Study design

An online cross-sectional questionnaire-based survey was conducted from January to March, 2022, and was approved by the Ethics Committee of Medical college, Wuhan University of Science and Technology (No.: WUST-21392). General public currently living in every region of China were recruited to collect the self-reported data using the survey link generated by the Questionnaire Star web survey software. A mixture of convenience and snowball sampling techniques was used for this survey. The survey link was initially forwarded to a convenience sample of enrolled students in Wuhan University of Science and Technology. In order to reach maximum samples in a short time, based on the snowball sampling technique (21), the responding students were requested to forward the survey link through online social networks to their fellow-students, family members, relatives and friends, etc., who were encouraged to continue to share the survey invitation with people they may know. The adults (aged 18 years and above) who can use social media apps such as WeChat and Tencent were invited to voluntarily and anonymously participate in this study after obtaining their informed consent.

### 2.2. Questionnaire

The initial draft of questionnaire was developed by the research team. The content validity, clarity, relevance and conciseness of questionnaire items were appraised by two public health researchers. The first 20 responding university students, whom the survey link was initially forwarded to, were asked to comment on volume, content, layout and intelligibility of the questionnaire, and their responses were not included in the final survey. After receiving feedback during the pilot phase, minor necessary corrections were made to finalize the questionnaire.

The final questionnaire consisted of four sections. The first section contained 5 questions regarding the demographic characteristics of responding Chinese residents, including gender, age, residence, education level and healthcare professional background. The second section included 3 questions related to the practice of consuming environmental friendly disinfectants in the household setting under current COVID-19 conditions. Concretely speaking, the participants were asked whether they paid special attention to the environmental certification label on the product, and only bought the environmental certified disinfectant products, whether they were using environmental friendly hand sanitizers, and whether their family was using environmental friendly disinfectants for household environmental disinfection. The third section was designed to measure the environmental knowledge levels regarding disinfectants among the participants. First, a question using a 5-point Likert scale, ranging from score of 1 (know nothing) to 5 (expert/know everything), were designed for the participants' self-evaluation of their own environmental knowledge levels pertaining to disinfectants. In addition, in order to capture the actual knowledge levels, the 5 objective knowledge questions with multiple-choice answers were designed, which were about the possible entrance routes for disinfectants to the environment, their potential environmental risks, human exposure hazards, the known environmentally friendly disinfectant components, as well as the contents about excessive disinfection in the Chinese national “Guideline for use of disinfectants (2020 version)” (22), respectively. The participants received score of 1 for each correct and complete answer. Otherwise they receive knowledge score of 0. The total objectively assessed environmental knowledge scores ranged from 0 to 5. In the last section of questionnaire, participants were asked 5 questions that covered the attitudes of Chinese residents toward the development, consumption and application of environmental friendly disinfectants, including four ones answered in a 5-point Likert-scale format ranging from “strongly disagree” (score of 1) to “strongly agree” (score of 5), and the other single-choice question to capture the participants' perceived barriers jeopardizing the usage intention regarding environmental friendly disinfectants. The Likert-scale-based questions were designed to assess the participants' attitudes about the threats of sharply increased disinfectant use triggered by COVID-19 to environment and ecosystem, the necessity of developing and marketing environmental friendly disinfectants, whether they were pleased to use the environmental friendly disinfectants, and whether they were more willing to buy and pay for environmental friendly disinfectants than common ones at the same price, respectively.

Using forward and reverse translations, the questionnaire was originally developed in English, then translated to Chinese and back translated to English. The final questionnaire in Chinese was administered to the participants, and required to be completed within 15 min. The Cronbach's α value and the Kaiser-Meyer-Olkin (KMO) measure of the final questionnaire were 0.801 and 0.796, respectively.

### 2.3. Statistical analysis

Data were entered into SPSS 26.0 for statistical analysis. Results were shown as mean ± standard deviation (SD) for quantitative data and numbers (percentages) for categorical data. The χ^2^ test was employed to compare categorical data. The one-way analysis of variance (ANOVA) with *post hoc* Tukey's honestly significant difference (HSD) analysis was used for comparison of quantitative variables. *P* < 0.05 or < 0.01 was considered statistically significant.

## 3. Results

### 3.1. Participants' characteristics

By the end of survey period, a total of 1,918 completed questionnaires were collected. Among them, 57 questionnaires were excluded because they were completed over the required time period (15 min) according to the on-line time recording in the Questionnaire Star platform. Therefore, 1,861 questionnaires were ultimately included in the survey analysis. The included participants' demographic information was shown in Table 1. Among these 1,861 Chinese residents, the gender distribution was approximately equal, with 869 (47%) males and 992 (53%) females. In terms of age groups, 642 participants were aged between 18 and 20, which accounted for 34% of the included residents. The next highest age group were those aged between 41 and 65, and the least well represented group were old people aged over 65 years. From a location perspective, nearly half (42%) of participants came from municipalities or provincial capital cities, and 22% lived in villages and towns. The overwhelming majority (81%) of participants had not the healthcare professional background. Half participants were undergraduates or bachelor's degree holders and 254 (14%) were postgraduates.

**Table 1:** Demographic information, practice of consuming environmental friendly disinfectants and environmental knowledge regarding disinfectants among participants (*n* = 1,861).

**Participant attribute**	**Number (%)**	**Participants who agreed or strongly agreed that they pay special attention to the environmental certification label on the product, and only buy the environmental certified disinfectant products**			**Participants who are using environmental friendly hand sanitizers**			**Participants whose family is using environmental friendly disinfectants for household environmental disinfection**			**Self-evaluated environmental knowledge score, Mean ±SD**	**Objectively assessed environmental knowledge score, Mean ±SD**
		**Number (%)**	χ**2**	***P-*** **value**	**Number (%)**	χ**2**	***P-*** **value**	**Number (%)**	χ^2^	***P-*** **value**		
**Gender**												
Male	869 (47)	125 (14)	11.076	0.001	133 (15)	0.365	0.546	78 (9)	0.543	0.461	2.43 ± 1.56	2.08 ± 1.94
Female	992 (53)	201 (20)			162 (16)			99 (10)			2.41 ± 1.88	2.15 ± 2.01
**Age**												
18–20 years	642 (34)	84 (13)	20.508	0.000	53 (8)	52.672	0.000	11 (2)	70.199	0.000	2.32 ± 1.40	2.09 ± 2.08
20–40 years	549 (30)	126 (23)			126 (23)			78 (14)			2.53 ± 1.93	2.14 ± 1.41
41–65 years	638 (34)	112 (18)			114 (18)			85 (13)			2.41 ± 1.89	2.13 ± 1.55
>65 years	32 (2)	4 (13)			2 (6)			3 (9)			2.66 ± 1.37	1.94 ± 0.83
**Residence**												
Municipalities or provincial capital cities	789 (42)	175 (22)	75.755	0.000	128 (16)	18.447	0.000	89 (11)	6.174	0.046	2.49 ± 1.45	2.16 ± 2.06
Other cities	669 (36)	139 (21)			129 (19)			60 (9)			2.31 ± 1.92	2.13 ± 1.72
Villages and towns	403 (22)	12 (3)			38 (9)			28 (7)			2.47 ± 1.86	2.01 ± 1.87
**Education level**												
Junior college or below	677 (36)	42 (6)	136.941	0.000	99 (15)	4.926	0.085	64 (9)	0.297	0.862	2.50 ± 1.77	2.06 ± 1.99
Undergraduate	930 (50)	188 (20)			144 (15)			91 (10)			2.32 ± 1.90	2.15 ± 1.49
Postgraduate	254 (14)	96 (38)			52 (20)			22 (9)			2.56 ± 1.94	2.17 ± 1.94
**Healthcare professional background**												
Yes	358 (19)	52 (15)	2.747	0.097	115 (32)	72.624	0.000	26 (7)	2.604	0.107	2.47 ± 1.33	2.19 ± 1.38
No	1,503 (81)	274 (18)			180 (12)			151 (10)			2.41 ± 2.02	2.10 ± 2.00

### 3.2. Practice of consuming environmental friendly disinfectants in the household setting under COVID-19 conditions

**Figure 1:**
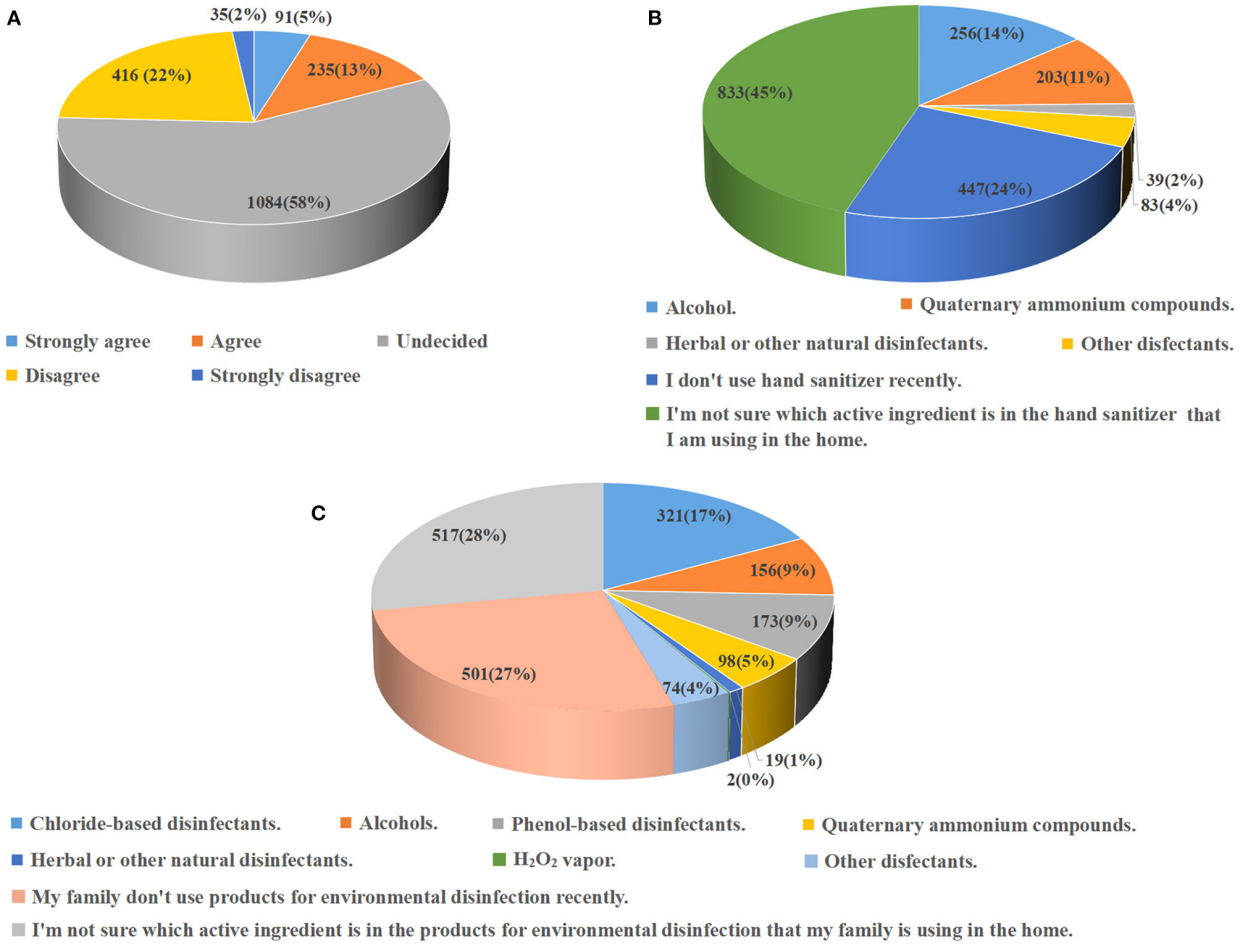
Practice of Chinese residents to use environmental friendly disinfectants in the household setting under COVID-19 conditions. **(A)** Participants' responses to the statement “*when I am choosing household disinfectant products under COVID-19 conditions, I pay special attention to the environmental certification label on the product, and only buy the environmental certified disinfectant products*”. The participants' perceived core active ingredients in the hand sanitizers **(B)** and products for environmental disinfection **(C)** currently used in their home.

Among the 1,861 participants, as shown in Figure 1A, when choosing household disinfectant products under COVID-19 conditions, only 326 (18%) responding Chinese residents agreed or strongly agreed that they paid special attention to the environmental certification label on the product, and only bought the environmental certified disinfectant products; while the most (58%) felt “undecided” about the environmental aspect of household disinfectant products. When answering the questions about core active ingredient in the hand sanitizer and product for environmental disinfection currently used in their home (Figures 1B, C), 45 and 28% of the participants claimed that they were not sure about the active ingredients in hand sanitizers and household products for environmental disinfection, respectively; about quarter (24 and 27%, respectively) residents did not use hand sanitizers and environmental disinfectants. In addition to these above, alcohols were believed as the most common core active ingredient in hand sanitizers, which was chosen by 256 (14%) participants; the second was quaternary ammonium compounds; and only 2% reported that they were using the hand sanitizers containing the herbal or other natural disinfectants as core active ingredients. As for the household products for environmental disinfection, the most popular core active ingredient was the chloride-based disinfectants. Based on the opinion that the environmental friendly disinfectants mainly include hydrogen peroxide-based, volatile, and natural ones (3, 5, 16, 17), only 16% (295) and 10% (177) of Chinese residents included in this survey were using environmental friendly disinfectants for hand sanitization and environmental disinfection, respectively, in the household setting under current COVID-19 conditions.

In terms of group-comparison, as shown in Table 1, gender, age, residence, and education level differences were found in the Chinese residents' attention to environmental certification label on household disinfectant products and buying intention of environmental friendly disinfectants (*P* < 0.01). Females, individuals aged 20–40 years, urban residents, and the higher educated attached greater importance to the environmental aspect of household disinfectant products. Regarding the practical use, significantly more individuals aged 20–40 years and urban residents were using environmental friendly disinfectants for hand sanitization and household disinfection during the survey period, compared with other age groups and rural residents (*P* < 0.05; *P* < 0.01). Moreover, participants having healthcare professional background tended to use the environmental friendly hand sanitizers containing alcohols or natural disinfectants (*P* < 0.01), but no profession difference was shown in the practice of using environmental friendly disinfectants for household environmental disinfection.

### 3.3. Environmental knowledge levels regarding disinfectants

The self-assessed and actual environmental knowledge levels regarding disinfectants were measured using a 5-point Likert scale and five close-ended questions, respectively. The mean self-assessed knowledge score for 1,861 responding Chinese residents was 2.42 ± 1.74 out of a total of 5; and mean score of actual knowledge was obtained as 2.12 ± 1.97 (total 5 points). When classifying the responding Chinese residents' knowledge levels according to their demographic characteristics, as shown in Table 1, there was no significant difference in either self-assessed or actual test score of the environmental knowledge about disinfectants by gender, age, profession, residence, or education level (*P* > 0.05).

Furthermore, we compared the environmental knowledge scores among participants reporting different practices of consuming environmental friendly disinfectants. As shown in Table 2, for the residents who attached their attention to environmental aspect of household disinfectant products and who were using environmental friendly disinfectants for household environmental disinfection, their self-evaluated knowledge scores were markedly higher (*P* < 0.01). The actual knowledge scores of participants who were using environmental friendly products for environmental disinfection were higher than those of participants who were not using this class of household disinfectants (*P* < 0.05). These data suggested that there might be a significant association between the practice of consuming environmental friendly disinfectants and the knowledge level among Chinese residents.

**Table 2:** Association between the practice of consuming environmental friendly disinfectants and the self-evaluated or objectively assessed environmental knowledge regarding disinfectants among participants (*n* = 1,861).

**Practice of consuming environmental friendly disinfectants**	**Self-evaluated environmental knowledge score**		**Objectively assessed environmental knowledge score**	
	**Mean** ±**SD**	***P-*** **value**	**Mean** ±**SD**	***P-*** **value**
**Participants opinion about the statement “*I pay special attention to the environmental certification label on the product, “and only buy the environmental certified disinfectant products*”**.				
• Agreed or strongly agreed (*n* = 326).	2.68 ± 1.63	0.002	2.25 ± 1.84	0.182
• Felt undecided, disagreed or strongly disagreed (*n* = 1,535).	2.36 ± 1.70		2.09 ± 1.99	
**Whether participant is using environmental friendly hand sanitizers?**				
• Yes (*n* = 295).	2.51 ± 1.82	0.318	2.16 ± 1.93	0.690
• No (*n* = 1,566).	2.40 ± 1.72		2.11 ± 1.98	
**Whether participant' family is using environmental friendly disinfectants for household environmental disinfection?**				
• Yes (*n* = 177).	2.74 ± 1.68	0.011	2.40 ± 1.81	0.046
• No (*n* = 1,684).	2.39 ± 1.75		2.09 ± 1.98	

### 3.4. Chinese residents' attitudes toward environmental friendly disinfectants

As shown in Table 3, the responding Chinese residents' overall attitudes toward the development, consumption and application of environmental friendly disinfectants were very positive. The threat to environment and ecosystems posed by disinfectants used to combat COVID-19 pandemic has been strongly agreed or agreed by a majority of (73%) participants. More than half (58%) of participants supported or strongly supported the development and market introduction of environmental friendly disinfectants. Encouragingly, most responding residents (63 and 67%, respectively) stated that they were very pleased to use the certificated environmental friendly disinfectants, and more willing to buy and pay for environmental friendly disinfectants than common ones at the same price.

**Table 3:** Respondents' attitudes toward environmental friendly disinfectants for household use in the post-pandemic period (*n* = 1,861).

**Survey question/statement**	**Responses, number (%)**				
	**Strongly agree**	**Agree**	**Undecided**	**Disagree**	**Strongly disagree**
1. The sharp rise in use of disinfectants to combat COVID-19 pandemic would cause an enormous threat to environment and ecosystems.	356 (19)	1,003 (54)	488 (26)	12 (1)	2 (0)
2. It is necessary to develop and market the environmental friendly disinfectants.	266 (14)	815 (44)	763 (41)	15 (1)	2 (1)
3. I am very pleased to use the environmental friendly disinfectants, if they have acquired quality and environmental certifications.	240 (13)	937 (50)	645 (35)	27 (1)	12 (1)
4. I am more willing to buy and pay for environmental friendly disinfectants than common ones at the same price.	251 (13)	1,012 (54)	544 (29)	39 (2)	15 (1)

**Figure 2:**
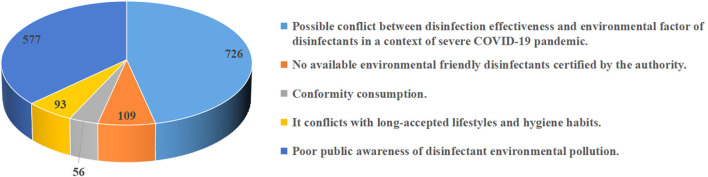
The most important perceived barrier jeopardizing the usage intention regarding environmental friendly disinfectants.

At the end of the questionnaire, the participants were asked the question, “*What is the most important perceived barrier jeopardizing your usage intention regarding environmental friendly disinfectants*?” (Figure 2). More than one-third (39%) of participants were worried about the “*possible conflict between disinfection effectiveness and environmental factor of disinfectants in a context of severe COVID-19 pandemic*”; another approximately one-third (31%) chose the barrier of “*poor public awareness of disinfectant environmental pollution*”. In addition, 22% of participants considered “*no available environmental friendly disinfectants certified by the authority*” as the most important barrier jeopardizing their own usage intention.

No significant attitude difference was found regarding each of sociodemographic and practice variables (*P* > 0.05).

## 4. Discussion

Currently, many disinfectants used in the household setting have been categorized as antimicrobials under pharmaceuticals and personal care products (PPCPs) (5), the latter of which have been accepted as an important class of emerging environmental contaminants and attracted increasing attention in recent years (5, 23). Especially for minimizing the environmental risks posed by pharmaceutical emerging contaminants, the green drug design under the concept of sustainable pharmacy has been believed as a critical component in the practical framework to integrate considerations for environmental fate and risk into the stage of new drug development (5, 24–27). The green drugs obtained through improving the environmental biodegradability of pharmaceutical molecules or developing plant-derived active compounds having minimal ecological impact have been generally considered as “eco-friendly” products and safer than their traditional counterparts (24–27). Similarly, the development and market introduction of environmental friendly disinfectants would act as a key route to minimize the risks posed by disinfectant emerging contaminants (2). To the best of our knowledge, the present study is the first to examine the practice of consuming environmental friendly disinfectants among potential customers, and to analyze the associated knowledge and attitude factors.

Our survey found that the current consumption of environmental friendly disinfectants in practice was extremely low among Chinese residents, which was evidenced by the results showing that only 18% participants paid special attention to the environmental certification label on the product, and only bought the environmental certified disinfectant products; 10–16% were using environmental friendly disinfectants for household use during the survey period. This finding was in line with another previous survey reporting that 12% of a total of 3,667 Chinese residents were concerned about the environmental impact of household disinfectant products (2). Nevertheless, we found the females, the young and middle-aged, urban residents, and the higher educated appeared to have relatively better practice of consuming environmental friendly disinfectants, suggesting that these population groups might be easier targets to market this emerging class of disinfectant products to. In addition, participants were asked three questions regarding their practice of using environmental friendly disinfectants, however, the acquirement of healthcare professional knowledge appeared to only be beneficial for pro-environmental behavior preference in consuming hand sanitizers. In fact, the alcohol-based hand sanitizers have been strongly recommended as the first-line of defense against SARS-CoV-2 and related viruses by the World Health Organization and national health authorities around the world (2, 22, 28), which are coincidentally identified to be environmental friendly because of high volatility (3). Therefore, whether individuals with healthcare professional background are more active in using environmental friendly disinfectants still needs further evaluation.

Of importance, we found a potential association between the practice of consuming environmental friendly disinfectants and the knowledge level among participants. Despite the fact that the overall average environmental knowledge levels regarding disinfectants were extremely low (2.12–2.42 out of a total of 5), participants having good practice of consuming environmental friendly disinfectants achieved significantly higher environmental knowledge scores. This finding was in accordance with the Information-Motivation-Behavioral Skills (IMB) model proposing that the awareness and understanding are generally prerequisite to willingness and behavioral change (29, 30). The poor environmental knowledge about disinfectants was also corroborated by the assessment results of practice showing a considerable proportion of participants had no opinion about the environmental aspect of disinfectant products and did not clearly know the active disinfectant ingredients for household use. Therefore, it could be speculated that a deep understanding might act as a driver for encouraging consumption and use of environmental friendly disinfectants in the household setting.

Attitudes toward environmental friendly products have been believed to significantly mediate the correlation of environmental concern and eco-literacy with the consumers' willingness to pay and payment behavior for environmental friendly products (31). Encouragingly, the present study found that, although the responding Chinese residents experienced poor practice of consuming environmental friendly disinfectants and lacked the related environmental knowledge, their attitudes toward the development, consumption and application of environmental friendly disinfectants were found to be very positive. In line with recent government stimulus and awakened pro-environmental awareness of the public in China (2, 32, 33), considerable participants were aware of the possible threat to environment and ecosystems posed by disinfectants used to combat COVID-19 pandemic, appreciated and supported the development and market introduction of environmental friendly disinfectants, and reported their willingness to use and pay for this emerging class of disinfectants, suggesting a high customer acceptance and a strong consumption behavioral intention of environmental friendly disinfectants in the market of household healthcare product.

When the perceived barriers jeopardizing the usage intention regarding environmental friendly disinfectants were explored, we found multiple barriers, including the concern about the disinfection effectiveness, poor public awareness, as well as limited availability of environmental friendly disinfectants, appeared to be the greatest concerns and might be partly reasons for the gap between intention and actual consumption behavior. Among them, “*possible conflict between disinfection effectiveness and environmental factor of disinfectants in a context of severe COVID-19 pandemic”* was considered as the most important barrier for the market introduction of environmental friendly disinfectants among Chinese residents. A previous study (34) mentioned that, when being faced with the threat of infectious disease, consumers often tend to increase preference for purchasing more familiar products because of the triggered emotional reaction of disgust and fear. Facing the COVID-19 pandemic being wreaking havoc, the purpose of buying disinfectant products for almost all the consumers is indisputably to effectively disinfect SARS-CoV-2 contaminated surfaces for control of COVID-19 (2). It is understandable that considerable participants were worried about the possible imbalance between the disinfection effectiveness and the environmental factors of disinfectants, since the fact that they are unfamiliar with the environmental friendly disinfectants, as an emerging class of disinfectants. Moreover, a significant portion of environmental friendly disinfectants is derived from plants and natural products (16, 17), which are usually perceived to be milder and safer than the chemical products (35). This stereotype might also cause the consumers' doubt about the disinfection effectiveness and performance of natural disinfectants. Therefore, the further development and market introduction of environmental friendly disinfectants exhibiting excellent disinfection activity against microorganisms, especially SARS-CoV-2, are of great importance.

Perhaps because of their emerging role in disinfection, the global scenario on the production and marketing of environmental friendly disinfectants is still lacking. However, it has been shown that, in addition to the alcohol-based hand sanitizers, other degradable disinfectants are currently playing trivial roles in the fight of COVID-19 according to the official recommendations (2, 22, 28). In fact, based on its effective disinfection for SARS-CoV-2 and minimal environmental impacts, hydrogen peroxide vapor treatment is an optimal choice for disinfecting surfaces, air, N95 respirators and other personal protective equipment (3). Accordingly, the on-demand production and marketing of such green chemicals for disinfection enable a circular economy model to sustainable epidemic prevention (3). As for the plant-derived natural disinfectants, a number of herbal-based hand sanitizer formulations were now commercially available in the public market in spite of the missing data about production (36). The positive attitudes of Chinese residents toward the development, consumption and application of environmental friendly disinfectants, shown in this survey, suggested the production of environmental friendly disinfectants would become more attractive and promising in China. During the post-pandemic economic recovery, the sustainable ecological development is being paid special attention and becoming a leading direction (37). Under the dual background of COVID-19 pandemic and sustainable development, the environmental friendly disinfectants might be facing a market opportunity. The popularization of environmental knowledge of disinfectants through differentiated publicity and education programmers should be taken to enhance the knowledge and interest of the population in the use of environmental friendly disinfectants. The wechat and other social media platforms could be used to market and report the green disinfectant products.

There are several limitations in this study. First, participants included in this survey was recruited using the snowball procedure, starting from a convenience sample of students enrolled in a university. This non-random sampling approach might influence representativeness of the sample. In addition, collecting the retrospective self-reported data in cross-sectional survey might result in recall bias, thus affect the reliability of the study.

## 5. Conclusions

In conclusion, this cross-sectional survey covering 1,861 responding Chinese residents firstly assessed the practices, knowledge, and attitudes of potential customers toward the environmental friendly disinfectants, an emerging and promising class of disinfectants for household use in the post-pandemic period. Results showed that 18% participants paid special attention to the environmental certification label on the disinfectant product, and only bought the environmental certified ones; only 16 and 10% were using environmental friendly disinfectants to sanitize their own hands and household environment, respective, suggesting majority of Chinese residents had poor practices of consuming environmental friendly disinfectants. Group-comparison showed that the females, the young and middle-aged, urban residents, and the higher educated appeared to have relatively better practice. In addition, the participants lacked environmental knowledge concerning disinfectants, but had favorable attitudes and strong buying intentions regarding environmental friendly disinfectants. Participants experiencing good practices of consuming environmental friendly disinfectants achieved higher knowledge scores. The “*possible conflict between disinfection effectiveness and environmental factor of disinfectants in a context of severe COVID-19 pandemic”* was considered as the most important barrier for the market introduction of environmental friendly disinfectants among Chinese residents. These findings suggested that it might be necessary to enhance the residents' knowledge levels of environmental issues posed by disinfectants based on public education campaigns, as well as to further develop the disinfectant products with both excellent disinfection activity and environmentally friendly attributes, and introduce them into the marketplace.

## Data availability statement

The original contributions presented in the study are included in the article/supplementary material, further inquiries can be directed to the corresponding author.

## Ethics statement

The studies involving human participants were reviewed and approved by the Ethics Committee of Medical College, Wuhan University of Science and Technology (No.: WUST-21392). The patients/participants provided their written informed consent to participate in this study.

## Author contributions

ZZ and JW designed the study. YT, ZZ, and WC drafted the initial manuscript. YT, WC, XH, and JW collected the data. YT, ZZ, WC, XH, and FW analyzed the data. YT, ZZ, WC, FW, XH, and JW reviewed and edited the manuscript. JW acts as a guarantor of the study. All authors reviewed and approved the final version of the manuscript.
